# The Relationship between Intimate Partner Violence and Family Planning among Girls and Young Women in the Philippines

**DOI:** 10.5539/gjhs.v8n9p121

**Published:** 2015-10-29

**Authors:** Laura Cordisco Tsai, Claudia Cappa, Nicole Petrowski

**Affiliations:** 1 George Mason University Department of Social Work, Fairfax, VA, USA; 2 United Nations Children’s Fund (UNICEF), New York, NY, USA

**Keywords:** domestic violence, contraceptive use, Responsible Parenthood and Reproductive Health Act, Southeast Asia

## Abstract

This study explored the relationship between intimate partner violence (IPV) and family planning among adolescent girls and young women in formal unions in the Philippines. Analyzing a sample (n =1,566) from the 2013 Philippines Demographic and Health Survey, logistic regression models were separately run for current contraception use and unmet need for family planning on recent physical violence (yes/no), recent sexual violence (yes/no), and recent emotional (yes/no). Findings revealed that the odds of using contraception were significantly higher among girls and young women who reported recent physical IPV (OR=1.84; 95% CI=1.13, 2.99; p<0.05) and sexual IPV (OR=2.18; 95% CI=1.17, 4.06; p<0.05). No significant relationship between recent emotional IPV and contraception use was found. Having an unmet need for family planning showed no significant relationship to IPV. The study adds to a growing body of literature revealing a positive association between IPV and contraception use. Findings hold implications for the provision of family planning services for adolescents and young women in response to the recent passage of landmark legislation pertaining to reproductive health in the Philippines, the Responsible Parenthood and Reproductive Health Act.

## 1. Introduction

The perpetration of violence at the hands of intimate partners is a serious public health and human rights concern for girls and women throughout the world. An estimated one in three adolescent girls aged 15 to 19 worldwide have experienced emotional, physical or sexual intimate partner violence (IPV) from their husbands or partners at some point in their lives (United Nations Children’s Fund (UNICEF), 2014). In the Philippines, adolescent girls and young women report the highest rates of IPV and physical violence during pregnancy of any age group in the country (Philippine Statistics Authority & ICF International, 2014). Little attention has, however, been given to the experiences of partner violence among girls and young women in the Philippines, with the majority of research pertaining to IPV from the country focusing on older women. Partner violence can have devastating consequences for the health, wellbeing and development of adolescents and young people. Partner violence has been associated with depression (Chandra, Satyanarayana, & Carey, 2009; Wong, Tiwari, Fong, Humphreys & Bullock, 2011), anxiety (Pico-Alfonso et al., 2006), post-traumatic stress disorder (Le, Tran, Nguyen, & Fisher, 2014), suicidality (Devries et al., 2011), and heightened risk behavior, such as substance use and high-risk sexual behavior (Coker, 2007). IPV can also be detrimental to the reproductive and sexual health of adolescents and young women, resulting in unwanted pregnancies, induced abortion, sexually transmitted infections, maternal morbidity and mortality, among others (Chambliss, 2008; Coker, 2007).

Globally, research pertaining to girls’ experiences of IPV and their family planning decisions is limited. Research with adult females from several countries has revealed that women who experience IPV have less control over their own fertility. For instance, research in the United States has found that women in violent relationships report fear of violence as a barrier to contraceptive use, with some studies indicating that abusive partners make decisions about contraceptive use as a means of exercising control over their partners (Williams, Larsen, & McCloskey, 2008). Women in the United States and Colombia who experience violence have been found more likely to report difficulties negotiating contraceptive use, more likely to report unintended pregnancies, and less likely to select their preferred method of contraception (Coggins, & Bullock, 2003; Pallitto, Campbell, & O’Campo, 2005; Pallitto & O’Campo, 2004; Williams, Larsen, & McCloskey, 2008). Similarly, another study conducted in Jordan suggested that women who have experienced IPV are more likely to have partners who have interfered with their efforts to avoid pregnancy (Clark et al., 2008). Women whose partners sabotage or are indifferent to their use of contraception are vulnerable to pregnancy and sexually transmitted infections (STIs) (Moore, Frohwirth, & Miller, 2010).

By contrast, other studies have found a positive association between IPV and contraceptive use. For instance, a study with married adult women in Cebu province in the Philippines found that ever having used modern contraception was positively associated with experiences of physical IPV among adult women (Hindin, & Adair, 2002). Another study of 6 Sub-Saharan African countries (Note 1) found that women who had experienced some form of IPV used contraception at a significantly higher rate than women who did not report any IPV (Alio, Daley, Nana, Duan & Salihu, 2009). In analyzing data from ten DHS national surveys from all world regions, Hindin, Kishor, and Ansara (2008) found that in 7 (Note 2) out of 10 countries, ever having used contraception was positively associated with IPV.

However, some studies have also found unique associations between different forms of IPV and family planning. For instance, a study analyzing DHS data of ever-married adult women in Jordan revealed that women who reported ever experiencing severe physical IPV were significantly less likely to use contraception, women who reported ever experiencing sexual IPV were more likely to use contraception, and emotional violence showed no relationship to contraception use (O’Hara, Tsai, Carlson, & Haidar, 2013). In contrast, Williams et al. (2008) found physical and emotional IPV to be negatively associated with contraception use and found no significant relationship between sexual IPV and contraception use.

These mixed findings underscore a general uncertainty in the literature regarding the directional, temporal, and causal relationship between IPV and contraception use. While the majority of empirical research indicates that contraception use and IPV are significantly associated, there are distinct findings to support: 1) IPV as negatively associated with contraception use, 2) IPV as positively associated with contraception use, 3) the relationship varying per type of IPV, and 4) the significance of any potential association as predicated on the temporal ordering of contraception use and IPV. Further, the vast majority of prior research on this topic has focused on the experiences of adult women; how these dynamics play out in the lives of girls and young women in formal unions has been understudied.

### 1.1 Philippines Context

The Southeast Asian region has seen a general decline in marriage and fertility in recent decades and an increase in age at first marriage (Jones, 2005; Jones & Yeung, 2014). Substantial variation across and between countries, however, remains (Williams, Kambalan, & Ogena, 2007). The Philippines is the only predominately Catholic country in Southeast Asia, with more than 80% of the population identifying as Roman Catholic. The Catholic Church maintains a very strong presence in political, social and cultural life in the Philippines (Austria, 2004). In prior decades, the Philippines held one of the lowest rates of adolescent marriage in Southeast Asia. However, by 2000, the Philippines had one of the highest rates of adolescent marriage in the region, after significant declines in other countries (Jones, 2011). One of the key determinants of age at first marriage among young women in the Philippines is their level of education, with increased education delaying age at first marriage (Abalos, 2014). A girl who marries early may find herself in a vulnerable position in relation to her partner and his family, as she may be more economically dependent than her unmarried peers and may be separated from other sources of social support (UNICEF, 2001, 2011). Further, early marriage is associated with higher rates of intimate partner violence among females (Le et al., 2014; Rahman, Hoque, Mostofa, & Makinoda, 2014). Divorce is, however, illegal in the Philippines, making it difficult for young women to leave violent partnerships.

Although early marriage commonly results in early pregnancy, the topic of contraception use among girls and young women in the Philippines is highly controversial. Abortion is illegal in the Philippines, and the use of modern forms of contraception is not only widely opposed, but also prohibitively expensive for households in poverty. Premarital sex is discouraged among youth – particularly for girls – and many young people remain uninformed about reproductive health services (Blanc, Tsui, Croft, & Trevitt, 2009; Mello, Powlowski, Juan, Nanagas, & Bossert, 2006; Upadhyay & Hindin, 2007). Social systems, such as schools, families, and health programs, have not traditionally provided the information necessary for Filipino youth to make informed decisions surrounding sexual and reproductive health issues (Upadhyay, & Hindin, 2007; Austria, 2004). Although fertility rates have dropped close to replacement levels in much of Southeast Asia, the Philippines maintains a fertility level comparable to that of the 1980s (Jones, 2013). Rates of teenage pregnancy in the Philippines are among the highest in Southeast Asia. Girls and young women report the lowest usage of contraception and having the highest unmet need for family planning services of all age groups in the Philippines (Huang Soo Lee & Cheng, 2012; Philippine Statistics Authority & ICF International, 2014). Adolescents with an unmet need for family planning are at risk for unintended pregnancies; some of these adolescents will resort to illegal (and often unsafe) abortions (Guttmacher Institute, 2015).

This paper meets a gap in the literature by exploring the relationship between experiences of intimate partner violence and the use of family planning methods among girls and young women in formal unions in the Philippines. To the authors’ knowledge, this is the first study to explore this subject among minors and young women using a population-based sample. The current study utilized the 2013 Philippines Demographic and Health Survey (DHS) data to address the following research question: Is the use of family planning significantly associated with intimate partner violence among adolescent girls and young women in the Philippines? The study hypothesis is that there is a significant positive relationship between IPV and use of family planning methods, since prior research in the Philippines has found a positive relationship between the two among adult women (Hindin, & Adair, 2002). As this study was based on publicly available data, institutional review board approval was not sought.

## 2. Methodology

### 2.1 Sample

The DHS encompasses a nationally representative sample of girls and women in the Philippines aged 15 to 49 years. The sample selection methodology was based on a stratified two-stage sample design, utilizing the 2010 Philippines Census of Population and Housing (CPH) as a frame. In this study, 14,804 households were successfully interviewed, representing a response rate of 99.4 percent. The DHS domestic violence module, entitled the ‘women’s safety module’ in this survey, was administered to 10,963 girls and women, with a response rate of 96.4 percent. For the current paper, data analysis was restricted to respondents aged 15 to 24 years who reported being in a formal union (either being married or living with a partner as if married). As such, the total sample size for this paper was 1,566 participants, including 1,112 respondents who participated in the women’s safety module (Philippine Statistics Authority & ICF International, 2014).

### 2.2 Measurement

Independent variables included experiences of physical, sexual and emotional partner violence in the past 12 months (recent IPV). In the DHS survey, IPV questions were based on the Revised Conflict Tactics Scale (Straus et al., 1996). Physical violence included pushing, shaking, throwing something at subject, slapping, twisting arm, pulling hair, punching or hitting with something that could hurt, kicking, dragging, beating up, choking, burning, and threatening and attacking with a knife, gun or other weapon. Sexual violence included: physically forcing girl/woman to engage in sexual intercourse or other sexual acts she did not want to engage in, forcing with threats or in any other way to engage in sexual intercourse or other sexual acts she did not want to engage in, and trying or attempting to force, persuade, or threaten girl/woman to engage in sexual intercourse or other sexual acts against her will. Emotional violence included humiliating girl/woman in front of others, threatening harm to self or someone the girl/woman cared about, and insulting the subject. Current use of contraception and an unmet need for family planning served as the dependent variables for the study. Current use of contraception included modern forms (e.g. intrauterine device, pill, condoms), traditional forms (e.g. withdrawal, rhythm method) and folkloric methods. An unmet need for family planning was defined as having an unmet need for birth spacing, an unmet need for birth limiting, and/or birth spacing or limiting failure.

As indicators of perceived gender roles and women’s status, we also included questions pertaining to girls’ and young women’s attitudes toward wife beating and decision making regarding contraception use. To understand their attitudes toward wife beating, respondents were asked if they approved of wife beating under a series of conditions, including the wife going out without husband’s permission, neglecting children, arguing with husband, refusing to have sexual relations with husband, and burning the food. To assess girls and women’s involvement in decision making regarding contraception usage, participants were asked: Would you say that using contraception is mainly your decision, mainly your husband/partner’s decision, or did you both decide together? Responses included mainly respondent, mainly husband, joint decision and other.

### 2.3 Data Analysis

Analysis was conducted using *Stata/IC*, version 11.0. We used chi-squared tests of independence to compare the age group of participants (15 to 19 and 20 to 24) by relevant socio-demographic characteristics and decision making regarding contraceptive use. Additionally, we utilized chi-square tests of independence to compare the percentage of girls and young women who reported using/not using contraception by attitudes toward wife beating and types of recent IPV experienced.

We conducted three logistic regression models to explore the relationship between current contraception use, recent physical IPV, recent sexual IPV, and recent emotional IPV among girls and young women in formal unions. We also ran three logistic regression models to explore the relationship between having an unmet need for family planning, recent physical IPV, recent sexual IPV, and recent emotional IPV. All of the logistic regression models controlled for the following covariates: participant’s age (continuous), marital status, highest level of education attended or completed (none/primary, secondary or higher), wealth quintile, residence (urban vs. rural), religion (Roman Catholic or other), respondent currently working (yes/no), age at first cohabitation with partner, total number of children, whether wife beating is ever justified by participant in any circumstance (yes/no), and the region of the Philippines in which the participant was residing at the time of the study.

## 3. Results

### 3.1 Characteristics of Study Sample

Out of 1,566 girls and young women aged 15 to 24 included in the study, 1,247 (80%) were aged 20 or above. Almost 20% of participants had only attended primary school or less, including 29% of those in the youngest age group. Girls aged 15 to 19 were significantly more likely than young women aged 20 to 24 to be in the poorest wealth quintile. Nearly three-fourths of participants reported being Catholic.

Young women aged 20 to 24 were significantly more likely than girls 15 to 19 to be married rather than cohabiting with a partner, with 50% of young women reporting being married compared to 24% of girls aged 15 to 19. On average, girls started cohabiting with a partner at the age of 16, while young women aged 20 to 24 started cohabiting with a partner at an average age of 19. Most respondents reported having an older partner, with an average age gap between respondents and their partners of 4.6 years. Among girls aged 15 to 19 years, the mean age of their partners was 22.9 years. The mean age of the partners of young women aged 20 to 24 was 26.8 years. However, 10.2% of all respondents reported having a partner that was at least 10 years older than them.

A total of 18% of participants were pregnant at the time of the study. Girls were significantly more likely to be pregnant at the time of the study than young women (p<0.000), with over one-fourth (26%) of adolescent girls reporting being pregnant during the study. The majority of participants (70%) already had one or two children at the time of the study. Less than half of participants reported using contraception (see Table 1).

**Table 1:** Socio-demographic characteristics of study sample (n = 1,566)

	Girls	Young women	Total	p-value
	15–19 years	20–24 years	15–24 years	
	n (%) or	n (%) or	n (%) or	
	*M (SD)*	*M (SD)*	*M (SD)*	
	n = 319	n = 1,247	n = 1,566	
*Highest level of education*				
Primary or less	92 (28.8)	215 (17.2)	307 (19.6)	< 0.000
Secondary	198 (62.1)	705 (56.5)	903 (57.7)	< 0.01
Higher	29 (9.1)	327 (26.2)	356 (22.7)	< 0.000

*Religion*				
Catholic	230 (72.1)	904 (72.5)	1,134 (72.4)	
Muslim	30 (9.4)	121 (9.7)	151 (9.6)	

Protestant	22 (6.9)	61 (4.9)	83 (5.3)	
Other	37 (11.6)	161 (12.9)	198 (12.7)	

*Wealth quintile*				
Poorest	103 (32.3)	319 (25.6)	422 (27.0)	p < 0.05

Poorer	82 (25.7)	321 (25.7)	403 (25.7)	
Middle	73 (22.9)	275 (22.1)	348 (22.2)	
Richer	38 (11.9)	220 (17.6)	258 (16.5)	p < 0.05
Richest	23 (7.2)	112 (9.0)	135 (8.6)	

*Residence*				
Urban	120 (37.6)	528 (42.3)	648 (41.4)	
Rural	199 (62.4)	719 (57.7)	918 (58.6)	

*Partner relationship*				
Married	77 (24.1)	622 (49.9)	699 (44.6)	< 0.000
Living with partner	242 (75.9)	625 (50.1)	867 (55.4)	< 0.000
*Age at first cohabitation*	16.3 (1.6)	18.5 (2.3)	18.1 (2.4)	< 0.000

*Average age of partner*	22.9 (4.4)	26.8 (5.3)	26.0 (5.4)	< 0.000

*Total number of children ever born*				
0	119 (37.3)	208 (16.7)	327 (20.9)	< 0.000
1-2	197 (61.8)	897 (71.9)	1,094 (69.9)	< 0.000
3-5	3 (0.9)	142 (11.4)	145 (9.3)	< 0.000

*Currently pregnant*	83 (26.0)	198 (15.9)	281 (17.9)	< 0.000

### 3.2 Girls and Young Women’s Empowerment

The majority of participants (81%) reported that they made decisions about their contraceptive use jointly with their partners (see Table 2). Roughly 1 in 8 girls and young women noted they made these decisions on their own, while 7% said their partners made the decision. Young women were significantly more likely to report joint decision making with their partners.

**Table 2:** Decision making regarding contraception use by age (n = 1,566)

	Girls	Young women	Total	p-value
	15 – 19 years	20 – 24 years	15 – 24 years
	n (%)	n (%)	n (%)
	n = 319	n = 1,247	n = 1,566
*Decision making regarding contraception use*				
Woman/girl	19 (17.0)	69 (11.3)	88 (12.2)	
Partner/husband	9 (8.0)	39 (6.4)	48 (6.7)	
Joint (respondent/partner)	82 (73.2)	500 (82.1)	582 (80.7)	p < 0.05
Other	2 (1.8)	1 (0.2)	3 (0.4)	

Almost one-fifth (18%) of participants indicated that wife beating was at times justified, with the most commonly reported reason being if the wife neglected the children (14%). Girls were more likely to report wife beating as justified than young women, but the difference was not statistically significant. As reflected in Table 3, girls and young women who reported that wife beating was justified under some circumstances were significantly less likely to use contraception.

**Table 3:** Attitudes toward wife beating by current contraception use (n = 1,566)

	Not currently using contraception (%)	Currently using contraception n (%)	Total n (%)	p-value
*Agree that wife beating is justified if the wife…*				
Goes out without telling husband	60 (7.1)	27 (3.7)	87 (5.6)	p<0.01
Neglects the children	126 (15.0)	91 (12.6)	217 (13.9)	
Argues with husband	45 (5.3)	22 (3.0)	67 (4.3)	p<0.05
Refuses to have sexual relations with husband	22 (2.6)	12 (1.7)	34 (2.2)	
Burns food	22 (2.6)	14 (1.9)	36 (2.3)	
In at least one of the above condition	167 (19.8)	108 (14.9)	275 (17.6)	p<0.05

### 3.3 Experiences of Violence and Use of Contraception

Exploratory data analysis revealed that 22% of girls and young women reported having experienced IPV in the past 12 months. Emotional violence was the most commonly reported form of violence, followed by physical IPV and sexual IPV. Less than half of girls and young women reported currently using some form of contraception, including 35% of girls and 49% of young women. Girls and young women who reported experiencing recent physical, sexual and emotional violence were significantly more likely to report using some form of contraception (see Table 4).

**Table 4:** Types of intimate partner violence among girls and young women by contraception use (n = 1,113)

	Not currently using contraception n (%)	Currently using contraception n (%)	Total n (%)	p-value
*Recent IPV*				
Any IPV	115 (20.14)	130 (24.0)	245 (22.0)	
Physical IPV	35 (6.12)	57 (10.5)	92 (8.3)	p<0.01
Sexual IPV	20 (3.50)	33 (6.1)	53 (4.8)	p<0.05
Emotional IPV	56 (9.81)	76 (14.0)	132 (11.9)	p<0.05

### 3.4 Logistic Regressions

Controlling for all aforementioned covariates, the odds of currently using contraception were on average significantly higher for girls and young women who reported recently experiencing physical IPV (Model 1 OR=1.84; 95% CI=1.13, 2.99; p<0.05). The odds of currently using contraception were also on average significantly higher for girls and young women who reported recent sexual IPV (Model 2 OR=2.18; 95% CI=1.17, 4.06; p<0.05). No significant relationship between recent experiences of emotional violence and current contraception use was found (Model 3 OR=1.47; 95% CI=0.98, 2.20; p=0.06), as reflected in Table 5. Across all three models, the odds of using contraception were significantly higher when girls and young women were married as opposed to cohabiting only; the odds of using contraception also increased significantly with each additional child they bore. None of the logistic regression models for unmet need for family planning on any of the types of IPV were significant (results not shown).

**Table 5:** Logistic regression models of current contraception use and intimate partner violence (n=1,113)

	Model 1			Model 2			Model 3		
		
	*OR*	*z*	*95% CI*	*OR*	*z*	*95% CI*	*OR*	*z*	*95% CI*
Recent physical violence	1.84*	2.47	(1.13, 2.99)						
Recent sexual violence				2.18*	2.44	(1.17, 4.06)			
Recent emotional violence							1.47	1.86	(0.98, 2.20)
Age	1.05	1.40	(0.98, 1.12)	1.05	1.53	(0.99, 1.13)	1.05	1.51	(0.98, 1.13)
Poorest wealth quintile	0.64	-1.45	(0.35, 1.17)	0.63	-1.50	(0.34, 1.15)	0.65	-1.38	(0.35, 1.20)
Poorer wealth quintile	0.89	-0.41	(0.50, 1.58)	0.88	-0.44	(0.49, 1.56)	0.91	-0.32	(0.51, 1.62)
Middle wealth quintile	0.96	-0.14	(0.55, 1.70)	0.95	-0.19	(0.54, 1.67)	0.96	-0.15	(0.54, 1.69)
Richer wealth quintile	1.12	0.36	(0.62, 2.03)	1.13	0.40	(0.62, 2.05)	1.15	0.47	(0.64, 2.09)
Urban	0.95	-0.34	(0.69, 1.30)	0.94	-0.40	(0.68, 1.28)	0.95	-0.32	(0.69, 1.30)
Catholic	1.10	0.54	(0.78, 1.54)	1.09	0.52	(0.78, 1.53)	1.10	0.54	(0.78, 1.53)
Completed secondary school or higher	1.36	1.64	(0.94, 1.96)	1.36	1.64	(0.94, 1.95)	1.34	1.57	(0.93, 1.93)
Wife beating justified (yes)	0.90	-1.13	(0.74, 1.08)	0.90	-1.08	(0.75, 1.09)	0.91	-0.99	(0.75, 1.10)
National Capital Region	3.47***	3.51	(1.73, 6.97)	3.60***	3.61	(1.79, 7.21)	3.67***	3.65	(1.83, 7.37)
Cordillera Administrative Region	1.30	0.64	(0.58, 2.93)	1.37	0.77	(0.61, 3.08)	1.38	0.78	(0.62, 3.11)
Ilocos Region	1.95	1.72	(0.91, 4.16)	1.96	1.74	(0.92, 4.19)	1.96	1.73	(0.92, 4.19)
Cagayan Valley	1.49	1.11	(0.74, 3.01)	1.53	1.18	(0.76, 3.08)	1.57	1.26	(0.78, 3.17)
Central Luzon	1.37	0.93	(0.70, 2.70)	1.45	1.08	(0.74, 2.84)	1.45	1.08	(0.74, 2.85)
CALABARZON	1.45	1.07	(0.73, 2.87)	1.47	1.12	(0.74, 2.91)	1.52	1.20	(0.77, 3.00)
MIMAROPA	0.72	-0.77	(0.31, 1.67)	0.72	-0.77	(0.31, 1.67)	0.72	-0.76	(0.31, 1.68)
Bicol	1.27	0.60	(0.59, 2.72)	1.29	0.65	(0.60, 2.77)	1.32	0.71	(0.61, 2.85)
Western Visayas	2.46*	2.43	(1.19, 5.08)	2.59*	2.58	(1.26, 5.35)	2.51*	2.49	(1.22, 5.19)
Central Visayas	1.61	1.26	(0.77, 3.38)	1.57	1.20	(0.75, 3.28)	1.64	1.31	(0.78, 3.43)
Eastern Visayas	2.62*	2.19	(1.11, 6.22)	2.62*	2.19	(1.11, 6.20)	2.63*	2.20	(1.11, 6.20)
Zamboanga Peninsula	0.93	-0.20	(0.45, 1.90)	0.97	-0.07	(0.48, 1.99)	0.97	-0.08	(0.48, 1.98)
Northern Mindanao	0.73	-0.76	(0.33, 1.64)	0.75	-0.71	(0.33, 1.68)	0.75	-0.71	(0.33, 1.67)
Davao	1.32	0.78	(0.66, 2.65)	1.38	0.90	(0.69, 2.75)	1.35	0.86	(0.68, 2.71)
SOCCSKSARGEN (Region XII)	1.21	0.52	(0.58, 2.52)	1.21	0.52	(0.58, 2.51)	1.24	0.57	(0.60, 2.57)
Autonomous Region in Muslim Mindanao	0.43*	-2.12	(0.20, 0.94)	0.44*	-2.10	(0.20, 0.94)	0.45*	-2.02	(0.21, 0.98)
Total number of children	3.02***	7.69	(2.28, 3.99)	3.03***	7.73	(2.29, 4.01)	2.97***	7.59	(2.24, 3.93)
Married	1.41*	2.27	(1.05, 1.89)	1.37*	2.10	(1.02, 1.84)	1.39*	2.18	(1.03, 1.86)

* p < 0.05

** p < 0.01

*** p < 0.001.

## 4. Discussion

The capacity of girls and young women in formal unions to control their fertility and choice of family planning methods is critical for their safety and development. The ability to plan the timing, number and spacing of their children without experiencing violence gives girls and young women freedom to plan the rest of their lives and prioritize their own education and growth. Unplanned pregnancies can limit girls’ and young women’s schooling and employment prospects (Blanc et al., 2009), deepening economic dependence upon their partners and potentially heightening their risk for experiencing violence (Heise, 2011; Postmus, Plummer, McMahon, Murshid, & Kim, 2012; Vyas & Watts, 2009). Unintended pregnancies are also associated with a range of adverse health outcomes for young mothers and infants, including delayed use of prenatal care, low birth weight, perinatal mortality, and postpartum complications (Pallitto et al., 2005). With over one-fifth of girls and young women in unions in this population-based sample reporting an unmet need for family planning (including more than one in four adolescent girls), the public health implications of increasing knowledge of family planning methods and access to contraception among youth in the Philippines are considerable.

The results of this study add to the existing body of literature that has revealed a positive relationship between various forms of IPV and contraception use (Alio et al., 2009; Ansara & Hindin, 2009; Fanslow et al., 2008; Hindin & Adair, 2002; O’Hara et al., 2013). While the reasons for this relationship cannot be ascertained from the current study, one possible explanation that has been suggested in the literature is that women in abusive relationships may attempt to prevent pregnancy because they do not want to bring a child into a violent family setting (Alio et al., 2009; Hindin & Adair, 2002). Regardless of the reasons behind the association, these findings suggest the importance of adequately addressing and responding to partner violence when providing sexual health services to girls and young women in the Philippines. In April 2014, the Philippines Supreme Court removed a ban on the Responsible Parenthood and Reproductive Health Act after a decade of fierce opposition (Guttmacher Institute, 2015). This landmark legislation mandates that the Filipino government increase access to reproductive health services, particularly among impoverished Filipinos, and provide sex education to public school students aged 10 to 19. The passage of this legislation creates a unique opportunity to address the sexual and reproductive health needs of adolescents and young people in the Philippines, as well as other inter-linked social issues such as partner violence.

Research in other contexts has shown that young adult women attending family planning clinics report higher rates of IPV in comparison to their peers (Miller et al., 2010). Prior studies have also found that clinic-based IPV assessment can be a critical step in identifying partner violence (Chang et al., 2003), highlighting the potential of health clinics providing family planning services to asses for IPV and link patients to broader IPV services. IPV screening could be incorporated as a component of sexual health care for girls and young women in the Philippines, as health facilities can provide a neutral venue for IPV service providers to gain access to girls and young women experiencing partner violence. Training should be provided to health care personnel to increase their capacity to identify the signs of partner violence, properly and safely screen for partner violence, and provide emergency and other referral services for girls and young women who disclose partner violence in health facility settings. It is also recommended that sex education sessions in public schools incorporate attention to gendered dynamics in youth sexual relationships and include education regarding consent, sexual negotiation, and partner violence. Health and sex education programs for youth should equip youth to reject violence as part of healthy relationships and to develop effective conflict resolution behaviors in relationships (Kaestle, & Halpern, 2005).

Study limitations include the self-reported and cross-sectional nature of the data. The potential for underreporting is a key concern in violence research due to social stigma, the sensitivity of the topic and concerns about privacy and safety (Pallitto & O’Campo, 2004; Shoultz et al., 2010). Given the cross-sectional nature of the study, it is unclear whether experiencing IPV leads girls to take steps to prevent pregnancy, whether utilizing contraception places girls at heightened risk for partner violence, or if the relationship is bi-directional. More exact temporal measurement is needed to speak to causal relationships. Further qualitative research is recommended in order to better define the association mechanisms between IPV and family planning for girls and young women in the Filipino cultural context.

Despite limitations, this study makes an important contribution to the global literature as it is the first study, to the authors’ knowledge, that has explored the intersection of partner violence and family planning specifically among girls and young women in the Philippines. Study findings highlight the importance of health care professionals understanding the connection between partner violence and family planning as sexual health services are expanded for youth in response to the Responsible Parenthood and Reproductive Health Act. While heightened efforts are needed to ensure that contraceptive services are provided to youth in an accessible, non-judgmental and confidential manner, it will also be critical for health care professionals to also understand the connection between IPV and the use of contraceptive methods. The rollout of this legislation provides a unique opportunity to not only increase access to sexual health services, but also to screen for IPV and provide necessary emergency and support services to girls and young women experiencing partner violence.
